# Perceived and Performed eHealth Literacy: Survey and Simulated Performance Test

**DOI:** 10.2196/humanfactors.6523

**Published:** 2017-01-17

**Authors:** Efrat Neter, Esther Brainin

**Affiliations:** ^1^ Department of Behavioral Sciences Ruppin Academic Center Emeq Hefer Israel

**Keywords:** eHealth, literacy, performance

## Abstract

**Background:**

Electronic health (eHealth) literacy of consumers is essential in order to improve information and communication technology (ICT) use for health purposes by ordinary citizens. However, performed eHealth literacy is seldom studied. Therefore, the present study assessed perceived and performed eHealth literacy using the recent conceptualization of health literacy skills.

**Objective:**

The aim of this paper was to examine the association between perceived and performed eHealth literacies.

**Methods:**

In total, 82 Israeli adults participated in the study, all 50 years and older, with a mean age of 67 (SD 11). Of the participants, 60% (49/82) were women and 72% (59/82) had a post-secondary education. The participants were first surveyed and then tested in a computer simulation of health-related Internet tasks. Performed, perceived (eHealth Literacy Scale, eHEALS), and evaluated eHealth literacy were assessed, and performed eHealth literacy was also recorded and re-evaluated later. Performance was scored for successful completion of tasks, and was also assessed by two researchers for motivation, confidence, and amount of help provided.

**Results:**

The skills of accessing, understanding, appraising, applying, and generating new information had decreasing successful completion rates. Generating new information was least correlated with other skills. Perceived and performed eHealth literacies were moderately correlated (r=.34, *P*=.01) while facets of performance (ie, digital literacy and eHealth literacy) were highly correlated (r=.82, *P*<.001). Participants low and high in performed eHealth literacy were significantly different: low performers were older and had used the Internet for less time, required more assistance, and were less confident in their conduct than high performers.

**Conclusions:**

The moderate association between perceived and performed eHealth literacy indicates that the latter should be assessed separately. In as much, the assessment of performed eHealth literacy in clinical settings should entail the structuring of tasks as well as shortening and automatizing the assessment.

## Introduction

Electronic health (eHealth) services have been rapidly expanding in many directions [1] yet connecting end-users to newly developed information and communication technologies (ICTs) and channeling patients to new products require an assessment of compatibility. End-user’s assessment is conveyed in the concept of eHealth literacy, defined as “the ability to seek, find, understand and appraise health information from electronic resources and apply such knowledge to addressing or solving a health problem” [2].

eHealth literacy includes the concept of health literacy [3,4] as well as traditional literacy and numeracy, information, media, computer, and scientific literacies, as presented in the Lily model [5]. Assessing users’ eHealth literacy has the potential to both align ICT technologies to consumers’ abilities to use them and empower the latter to fully participate in health-related, knowledge-based, decision-making [5]. However, eHealth literacy has been mostly assessed with the self-report eHealth Literacy Scale (eHEALS) measure developed by Norman and Skinner (2006). The eHEALS taps perceive skills [6-10] using a questionnaire, rather than the actual performance examination of eHealth literacy levels, mostly due to time and expense considerations [11]. While eHealth literacy was assessed mainly through self-reports, health literacy was assessed and found to be associated by both self-reports and performance tests (for reviews see [12-14]). Considering the advantage of employing a short measure for the assessment of eHealth literacy, information on the association between perceived and performed eHealth literacy is warranted. A related interesting question is whether eHealth literacy differs from digital literacy only in terms of content. Thus, a distinction between digital literacy and eHealth literacy skills should be examined.

Though the association between perceived and performed digital literacy has been extensively examined in several studies (for a review see [15]), few studies delved into the issue in the health context [10,11]. The most comprehensive set of studies on digital and eHealth literacy skills was carried out in the Netherlands [11,16-18]. These studies employed a taxonomy of health-related Internet skills, based on the authors’ digital taxonomy, consisting of medium-related skills (eg, operating a browser and navigating the Internet) and content-related skills (locating information and making use of it). The findings were consistent in locating deficiencies in skills, mostly in accessing information and making use of it, thus limiting users’ taking full advantage of the resources the Internet avails. The only study comparing perceived (eHEALS) and performed health-related Internet skills [10] found that the correlations between eHEALS and successfully completed tasks on an Internet skills performance test were weak and non-significant. These findings are somewhat surprising, considering the assumption that subjective and objective skills are theoretically related concepts different in their measurement tools; indeed, subjective and objective numeracy are highly correlated (about *r*=.60; [19]). As consumers gain more experience in Internet use for health purposes [20], it is possible that perceptions of skills and actual performance become more aligned, if they are measured accurately.

The current study aimed at examining the association between the eHEALS as a perceived measure of eHealth literacy and eHealth literacy performance on both digital skills and content-related health Internet skills. Health Internet skills were conceived in terms of the following recent conceptualization on health literacy [14]: (1) accessing, defined as “the ability to seek, find, and obtain health information” (similar to “locating” in van Deursen and van Dijk’s typology [17]); (2) understanding, defined as “the ability to comprehend the health information that is accessed;” (3) appraising, defined as “the ability to interpret, filter, judge and evaluate;” and (4) applying, defined as “the ability to communicate and use the information to make a decision to maintain and improve health.” The appraise and apply skills are similar to “making use” in van Deursen and van Dijk’s typology [17]. All these components relate Web 1.0 tasks. The Web 2.0 skill of generating new information was added to the performance test [21]. Furthermore, besides examining the successful accomplishment rate on the simulated tasks, the study also explored the process of accomplishing these tasks (eg, the confidence and motivation of participants), as perceived by the researchers and the amount of assistance required to complete the simulated tasks.

The following research questions were examined in this study: (1) Is successful completion rates of a task higher for relatively simple skills such as accessing and understanding health information and lower for appraising and applying? Is generating new materials the least successful task? (2) Is there an association between perceived and performed eHealth literacy, both at the overall skill level and between the components of the skills? (3) Is there a negative association between assistance provided in the performance tasks and skill level, both perceived and performed? (4) What are the associations between performed eHealth literacy and background characteristics (eg, age, gender, education, income, perceived health, and experience with the Internet)?

## Methods

A telephone survey and a face-to-face computer simulation (performance test) were conducted. The following sections describe participant recruitment, data collection, the tasks participants were asked to perform, and data analysis.

### Participants

Participants were recruited by a nationally representative random-digital-dial telephone household survey of Israeli adults aged 50 years and older. Calls were placed to 1206 residential households of whom 603 agreed to be interviewed, representing a 50.00% response rate and a sampling error of 2.04%. As there were only 206 participants (34.2%, 206/603) who used the Internet for health purposes in the representative sample, the sample was augmented by an additional 236 individuals (50 years or older who used the Internet for health purposes), resulting in 442 Internet users. Interviews were conducted in Hebrew, Arabic, and Russian by professional interviewers who went through a special training session to familiarize themselves with the questionnaire's terminology. The interviewers conducted the telephone survey using computer-assisted telephone interviewing software. At the end of the survey, participants who used the Internet for health purposes were asked whether they would be willing to participate in a second phase of the study. Those who agreed (22.9%, 101/442) were asked to provide contact information.

All 101 survey participants who agreed to participate in the second stage of the study were contacted and 28 (27.7%, 28/101) agreed to take part in the simulation and its recording. An additional 54 participants were recruited in a snowball fashion, using a selective quota to reach a sample as close as possible to the representative survey sample regarding gender, age, education, chronic medical conditions, and income, resulting in a total 82 participants who completed both the survey and the performance simulation (Table 1).

**Table 1:** Participant demographics in the simulation (n=82) and the representative samples (n=223).

Variable		Simulation	Representative sample
Age (years), mean (SD)		66.95 (11.62)	60.96 (8.54)
Gender (women), n (%)		49 (60%)	138 (61.9%)
Ethnicity (Jewish), n (%)		68 (83%)	201 (90.5%)
Chronic conditions, n (%)		35 (43%)	87 (39.0%)
SRH^a^, mean (SD)		3.08 (0.75)	3.30 (0.76)
**Education, n (%)**			
	Elementary to high school	21 (26%)	59 (26.5%)
	Post high school	59 (72%)	162 (72.6%)
Average income and above, n (%)		36 (53%)	118 (52.9%)
Internet experience (years), mean (SD)		12.16 (6.04)	10.17 (6.41)
Perceived eHealth^b^ literacy^c^, mean (SD)		3.17 (0.93)	3.12 (0.82)

^a^SRH: self-rated health.

^b^eHealth: electronic health.

^c^Perceived eHealth literacy measured on a scale from 1 to 5.

### Procedure

The survey took place first. Respondents to the telephone survey who agreed to be later contacted for the second phase of the study were tested in their homes. Participants who were recruited via snowball were also first contacted by telephone, followed by the survey administration and then the home test. The survey took about 30 minutes to complete whereas the performance simulation took approximately 1.5 hours to complete. The simulation was carried out on a portable computer connected to a cellular modem and was recorded by a TechSmith Morae Recorder, version 2.2. This approach controlled for quality of the hardware, software, and Internet connection, and ensured that the setting was similar for all participants. The advantage of conducting the simulation at the participant's home is that they were in a familiar location; however, the shortcoming is that they were required to use a computer that was configured differently from the device they ordinarily used, which may have affected their performance.

The telephone survey was conducted between December 2013 and March 2014. The computer simulations were carried out at the participants' homes between May 2014 and April 2015 and all participants signed an informed consent form and indicated their preferred language in the simulation. The participants who were recruited through the snowball technique responded to the telephone survey a couple of days prior to performing the face-to-face computer simulation. Participants were given a sequence of 15 assignments one at a time. Although there was a time frame allocated for each assignment, participants were not aware of it. When they hesitated or had difficulties completing tasks, the researcher helped them to complete the task and move on to the next. The researcher documented every instance that assistance was provided.

### Measurements

#### Perceived Electronic Health Literacy

Perceived eHealth literacy was measured by the eHEALS tool [5]. The scale is comprised of 8 items on a 5-point Likert scale from 1 (strongly disagree) to 5 (strongly agree). The scale was previously translated to Hebrew [9] and in a recent confirmatory factor analysis was found to be comprised of two factors: accessing and appraising [22].

#### Performed Digital and Electronic Health Literacy

Performed digital and eHealth literacy were measured through the completion of 15 computerized simulation tasks. The tasks were adapted from previous work [10,16-18,23,24] to the local context by conducting qualitative interviews and observations (eg, once a task was developed, it was run on 10 participants to assess acceptability, comprehension of instruction, and completion time). The tasks assessed digital skills and the health literacy skills used in Sorensen’s [14] typology of health literacy including accessing, understanding, appraising, applying, and generating information (see Multimedia Appendix 1 for the specific tasks and Multimedia Appendix 2 for the coding scheme of the tasks by skill type, specifying digital skills and eHealth literacy skills [11,25]). Only one task was allotted to the generating skills, as few people in this age group reported engaging in Web 2.0 activities in our focus groups, in the current survey, and in other surveys conducted at the time adjacent to the planning of the simulation [26]. A time frame was allocated to each task (Multimedia Appendix 1). Tasks were registered as “completed independently” or “not completed” by the researcher during their administration and upon reviewing the recorded performance. A second evaluation of recorded performance was conducted by a different researcher, and in cases of disagreement, a third researcher overruled. The time needed to perform the tasks was registered both by the researcher and by the recording software.

### Researcher’s Observations

A researcher performed a detailed and an overall observational judgment on each participant’s performance. The observational judgments pertained to the participants’ motivation to carry out the tasks, confidence, and proficiency level. All observational evaluations ranged from 1 (poor) to 5 (good). The observational evaluations were carried out both immediately after the completion of the tasks and later on the recorded performance. Two such observational evaluations were carried out on each performance, and in cases of disagreement a third observational evaluation took place.

### Assistance Evaluation

Once the time limit for task completion elapsed or a participant said she/he was about to give up on the task, participants were offered assistance. The researchers evaluated the amount of assistance given to participants and the assistance was summed across digital aspects (ie, medium-related, van Deursen and van Dijk’s typology, range 0 to 29), and health aspects (ie, content-related in terms of van Deursen and van Dijk’s taxonomy, range 0 to 16).

### Background Variables

Demographic and background variables related to health and Internet use (eg, age, gender, education, income, perceived health, and experience with the Internet) were documented as part of the survey.

### Data Analysis

First, the data for basic descriptive statistics for the key variables of background information, perceived and performed eHealth literacy was analyzed. Second, a series of bivariate tests were conducted to assess the association between the key variables of perceived and performed eHealth literacy and also with assistance provided. The participants were then divided into two groups, based on their performed eHealth literacy, and their scores on perceived eHealth literacy, amount of assistance provided, evaluated performance, and background characteristics compared using the analysis of variance (ANOVA) procedure. All analyses were carried out using SPSS Statistics, version 23.0 [27].

## Results

### Characteristics of Participants

Characteristics of the simulation sample and the survey representative sample are presented in Table 1. The simulation sample was 60% (49/82) women, with a mean age of 66.95 (SD 11.62), and 83% (68/82) Jewish. About half of the participants reported chronic medical conditions, 72% (59/82) had post secondary education, and 53% (36/82) described their income as average and above. Participants’ average length of time using the Internet was 12.2 years and they perceived their eHealth literacy level as moderate with mean of 3.17 (SD 0.93) on a 1 to 5 scale. Table 1 also presents the data on the characteristics of Internet users for health purposes from the representative sample. It can be seen that the simulation participants were older, less of Jewish ethnicity, reported similar income, and had more years of experience using the Internet, the latter possibly reflecting self-selection of participants more experienced and skilled in using the Internet.

### Performed Electronic Health Literacy and Its Association With Demographic Attributes

Performance in the 15 tasks comprising the simulation was grouped according to skill type (digital literacy, accessing, understanding, evaluating, applying, and generating eHealth information). The descriptive statistics on performance and success rate in completing each skill type and the descriptive statistics for perceived eHealth literacy are shown in Table 2. It can be seen that the simpler the skill type, the higher the successful completion rate was. For example, 83% (10/12) of tasks involving accessing were completed successfully, as opposed to only 58% (2.3/4) of the tasks involving applying information. In addition, success rates in digital literacy are similar to success rates in the eHealth skills of accessing and understanding but higher than the other skills.

In order to examine the concurrent validity of performed eHealth literacy, participants were assigned to two groups based on their mean score obtained on the performed eHealth literacy scale, similar to an analysis carried out by van der Vaart et al [10]. We used the median score of the scale in this sample (median 28 on a range of 0 to 35) to create two groups: those with a high mean performed eHealth literacy score (median 29 or greater); and those with a low mean performed eHealth literacy score (median less than 29). The demographic comparison between the two groups is presented in Table 3. Individuals in the low performance group had a mean age of 71.68 (SD 11.84), significantly older than in the high performing group, who had a mean age of 61.69 (SD 8.89) (F_1,74_=16.96, *P*<.001, eta square=0.186). In addition, they also had significantly fewer years of experience using the Internet with mean values of 10.54 (SD 5.81) and 14.13 (SD 6.14), respectively (F_1,74_=7.23, *P*=.009, eta square=0.085). They reported marginally significantly less education (F_1,80_ = 3.29, *P*=.074, eta square=0.039) and perceived themselves as marginally significantly less healthy than the high eHealth performing group with mean values of 2.95 (SD 0.93) and 3.36 (SD 0.89), respectively (F_1,74_=2.99, *P*=.088, eta square=0.036). There were no significant differences between the high and low eHealth literacy performance groups in perceived income (F_1,66_=1.25, *P*=.268, eta square=0.019) and the number of chronic medical conditions (F_1,66_ = 0.22, *P*=.642, eta square=0.003), nor were there differences in the gender distribution between the groups, for example 43% (17/40) men and 58% (23/40) women in the high performing group (χ^2^_1_=0.2, *P*=.684).

**Table 2:** Descriptive statistics of tasks by skill type (n=82).

		Range	Mean (SD)	Success rate^a^, %
Performed digital skills		0-35	29.70 (6.43)	71
**Performed eHealth^b^** **literacy**				
	Access	0-12	9.98 (2.69)	83
	Understand	0-10	7.34 (3.12)	73
	Appraise	0-8	5.05 (2.54)	63
	Apply	0-4	2.28 (1.51)	57
	Generate	0-1	0.46 (0.50)	46
	Overall	0-35	25.11 (9.58)	71
**Perceived eHealth literacy**				
	Access	1-5	3.36 (0.95)	N/A
	Appraise	1-5	2.83 (0.94)	N/A
	Overall	1-5	3.03 (0.85)	N/A

^a^Success rate determined using the mean value.

^b^eHealth: electronic health.

**Table 3:** Scores for the low (n=40) and high performed (n=42) eHealth literacy groups in background attributes, perceived electronic health literacy, assistance, and evaluations by observers.

Variable		Low, mean (SD)	High, mean (SD)	F/χ^2^_1_	*P* value	Eta square
**Background attributes**						
	Age	71.68 (11.84)	61.69 (8.89)	16.96	<.001	0.186
	Gender, n (%) women	26 (62)	23 (58)	0.17	.684	0.002
	Education^a^	3.93 (1.64)	4.50 (1.16)	3.29	.074	0.039
	Income^b^	2.55 (1.18)	2.89 (1.33)	1.25	.268	0.019
	Perceived health^c^	3.00 (0.91)	3.35 (0.92)	2.99	.088	0.036
	Chronic conditions, n	1.57 (0.70)	1.50 (0.68)	0.22	.642	0.003
	Internet use, years	10.54 (5.81)	14.13 (6.14)	7.23	.009	0.085
**eHealth^d^** **literacy**						
	Perceived eHealth literacy	2.67 (0.70)	3.39 (0.85)	16.59	<.001	0.174
	Assistance in digital skills^e^	8.84 (6.21)	3. 98 (4.90)	15.41	<.001	0.161
	Assist health content^f^	3.79 (3.16)	3.55 (4.35)	0.08	.779	0.001
**Evaluations^g^**						
	Skill	2.24 (0.79)	3.48 (1.01)	38.23	<.001	0.323
	Confidence	2.62 (0.96)	3.40 (0.98)	13.24	<.001	0.142
	Motivation	3.07 (0.89)	3.45 (1.01)	3.23	.076	0.039

^a^Education scored on a scale from 1 to 6.

^b^Income scored on a scale from 1 to 5.

^c^Perceived health scored on a scale from 1 to 5.

^d^eHealth: electronic health.

^e^Assistance in digital skills scored on a scale from 0 to 29.

^f^Assistance in health content scored on a scale from 0 to 16.

^g^Evaluations scored on a scale from 1 to 5.

### Performed, Perceived, and Evaluated Electronic Health Literacy

Pearson correlations between overall perceived and overall performed eHealth literacy was computed, as well as correlations between the perceived eHEALS factors of accessing and appraise in both modalities (Table 4). The correlation between overall perceived and performed eHealth literacy was *r*=.34 (*P*<.01), and a similar association was found between performed digital literacy and perceived eHealth literacy (*r*=.31, *P*=.002). The correlation between the perceived access factor was significant with performed skills of accessing, understanding, appraising, and applying (*r* ranged from .32 to .49, *P* values <.05) and the least with performed skill of generating (*r*=.22, *P*=.023). The correlation between the perceived appraise factor was significant with all performed skills (*r* ranged from .21 to .25, *P* values <.05) except generating (*r*=.17, *P*=.060). Generating information also correlated the least with all other performed skills and overall performance.

**Table 4:** Inter-class correlations between performed and perceived tasks (n=82).

Digital skills		Inter-class correlations								
		1	2	3	4	5	6	7	8	9
**Performed eHealth^a^** **literacy**										
	Access	.89^b^								
	Understand	.80^b^	.88^b^							
	Appraise	.69^b^	.79^b^	.93^b^						
	Apply	.64^b^	.74^b^	.81^b^	.80^b^					
	Generate	.49^b^	.53^b^	.58^b^	.58^b^	.68^b^				
	Overall	.82^b^	.92^b^	.98^b^	.94^b^	.88^b^	.65^b^			
**Perceived eHealth literacy**										
	Access	.34^b^	.32^b^	.39^b^	.49^b^	.36^b^	.22	.41^b^		
	Appraise	.24^c^	.21^c^	.21^c^	.24^c^	.25^c^	0.17	.24^c^	.61^b^	
	Overall	.31^b^	.28^c^	.31^b^	.37^b^	.33^b^	0.21	.34^b^	.84^b^	.94^b^

^a^eHealth: electronic health.

^b^Significant at .01.

^c^Significant at .05.

Participants in the low and high performed eHealth literacy groups were compared in terms of their perceived eHealth literacy score, the amount of help they received (digital and content), and the researchers’ judgment on motivation, skill, and confidence (Table 3). Participants low in performed eHealth literacy were significantly lower in perceived eHealth literacy, with a mean value of 2.67 (SD 0.70) than participants in the high performed eHealth literacy group whose mean value was 3.39 (SD 0.85) (F_1,79_ = 16.59, *P*<.001, eta square=0.174). Participants in the low performed eHealth literacy group were also granted more assistance, but only in the digital aspect of the tasks (F_1,79_ = 15.41, *P*<.001, eta square=0.161) and not on the health content aspect (F_1,79_ = 0.08, *P*=.779, η^2^=0.001) of the tasks. Participants in the low performed eHealth literacy group were consistently evaluated as significantly lower in skill (F_1,79_ = 38.23, *P*<.001, eta square=0.323), confidence (F_1,79_ = 15.41, *P*<.001, eta square=0.161), and marginally significant in motivation (F_1,79_ = 3.23, *P*=.039, eta square=0.089) by the researchers, compared with the high performing eHealth literacy group.

### Provision of Assistance and Skill Level

Pearson correlations between assistance provided for digital and eHealth content tasks and perceived and performed eHealth literacy were computed. There was a positive association (*r*=.67, *P*<.001) between the two kinds of assistance, so that the more assistance one was given on digital tasks the more assistance they were also given on the eHealth content tasks. Assistance on digital aspects was negatively associated with both perceived (*r*=-.41, *P*<.001) and performed score (*r*=-.34, *P*<.001) assessments suggesting that the more one was given assistance on digital aspects of tasks the lower the performed score and the lower the perceived skill. However, assistance on eHealth content was negatively associated with perceived eHealth literacy (*r*=-.25, *P*=.023) while not significantly associated with performed eHealth literacy (*r*=.07, *P*=.529).

## Discussion

### Principal Findings

The current study is unique in that it examines facets of eHealth literacy using different assessments (perceived, performed, and evaluated). Perceived eHealth literacy was assed using the eHEALS tool, whereas the performed eHealth literacy assessment was built on methodology and materials developed previously [10,21], while using the conceptualization of skills developed recently in the realm of health literacy. Evaluated eHealth literacy was carried out by two trained researchers, both during the simulation and subsequently, the latter using participants’ recorded performance (recording available through the software). Finally, the study also recorded and analyzed the amount of assistance provided to participants.

The study has several important findings. First, the more complex the skill (eg, applying information as opposed to accessing information), the lower the successful completion of tasks. Successful completion rates thus created a gradient made of accessing, understanding, appraising, applying, and generating information. The skill of generating information (eg, writing in a health forum) is of special interest since the success rates in this task were very low; however, it is unclear whether the task is more cognitively taxing or merely an unfamiliar activity for people in this age group.

The second and main finding of this study is that perceived and performed eHealth literacy is significantly associated with each other, though to a moderate degree. The finding suggests that people make a reasonable, though not accurate, evaluation of their skill level. The significant association is in line with findings on perceived and performed numeracy [19], though the size of the correlation is smaller in the case of eHealth literacy and could result from murkier standards on the skill. The only other identical examination in the literature is in a study by van der Vaart et al [10], where the associations were also positive yet lower, ranging from non-significant to marginally significant. Though the tasks employed in this study were modeled after the previous works of van der Vaart et al [10,21], with necessary adjustments to the health literacy typology [14] and to the Israeli context, the association between the same construct in two assessment modes was higher in the current study. This could be attributed to several differences in context between the studies. The current study had a more restricted sample age; the higher correlation between performed and perceived eHealth literacy may be partially attributed to older adults' relatively accurate judgments of their performance level. Indeed, van Deursen [11] has found that compared to younger participants, older participants select more relevant and more reliable resources, suggesting that in our study older users' eHealth literacy judgments were more reliable. In addition, participants in our current study were not rewarded for their time and effort financially, as opposed to van der Vaart et al’s study [10], and our study was conducted in the participants’ homes (rather than in a higher education institution) allowing for more comfort. Finally, assistance was provided to participants who experienced difficulties in completing various tasks. These differences in context could have affected the results in unforeseen ways.

A third finding is that participants who performed low and high in performed eHealth literacy were different from each other in other aspects reported in this study (ie, assistance, motivation, confidence, perceived skills, and background characteristics), re-iterating previous findings on the digital divide in the health domain [9,11]. Interestingly, the difference between the high and low performing groups in evaluated motivation was only marginally significant and its effect size was the lowest among the evaluations of skill and confidence, suggesting that it could be possible for individuals to upgrade their skills. Indeed, Norman and Skinner [5] viewed eHealth literacy as a malleable process that evolves all the time and not as a static attribute.

### Strengths and Limitations

The study possessed several strengths. First, it assessed eHealth literacy through actual performance, not relying on self-perceived assessment. It thus joins few works [10,11,21] in the field of health, possibly due to the arduous endeavor in terms of time and expenses [11]. Second, its sample is relatively big, considering the focus on performance. Third, the study augmented the perceived and performed assessment by a researcher’s evaluation. These evaluations went beyond performance to address confidence and motivation, hitherto not included in previous such work. The evaluations were carried out both immediately after the performance by one researcher and on the recorded performance by a second researcher, and in cases of disagreement, by a third researcher.

The study has also several limitations. First, the sample is age-skewed to older adults, from 50 years and older. Results could be somewhat different, especially in terms of successful completion rates of task, among a heterogeneously aged sample. Second, the fact that participants were recruited on a voluntary basis implies that they might already have been more interested in using the Internet and searching for information, which could have influenced the results. In addition, the snowball recruitment of some of the participants may have contributed to the relative homogeneity of the sample (eg, overrepresentation of older participants in the simulation, compared to the survey). Third, the skill of generating was assessed with only one task and future studies will probably enlarge the assessment of this skill in view of the increased prevalence of social media, the different interactive competencies called for [21], and as emerged from the data, the gap in skill level between generating and all other skills. Indeed, generating appears to be a unique skill, even during the age of social media; the other skills measured (ie, accessing, understanding, appraising, and applying information) apply to social media just as they apply to other sources in the Internet and offline inter-personal interactions. Fourth, all the tasks in the simulation were in the participants’ primary language in accordance with their preference (Hebrew, Arabic, and Russian); hence, participants were not challenged with content in a non-native language. Future studies, especially those conducted in locations with limited Internet content in a native language, could include performance section where participants are confronted with content not in their primary language. Fifth, the digital device used throughout the simulation was a laptop computer. As many people nowadays access the Internet via their mobile phones [28], where the operational skills needed are different (eg, using buttons, curser, clicking), future studies could add mobile health skills as well.

### Future Advances

Performed eHealth literacy was assessed laboriously in this study: the simulation took about 1.5 hours and a similar amount of time was required to code and evaluate the performance of a single person. This duration is clearly impractical in clinical settings. This calls for the development of a computerized, tailored test for performed eHealth literacy. The results of the present work indicate what this future tool could look like. Specifically, the moderate association between perceived and performed eHealth literacy, the high completion rates of accessing tasks concurrent with low variance, and the low completion rates in the generating task point to several attributes. First, the test needs to be short so that it can be applied in clinical settings. Hence, it could be adaptive so that performance determines the next task which saves time in measuring items an individual is likely to succeed in. Second, the test could contain a few perceived eHealth literacy items; perceptions take little time to measure and in this case are indicative of performance, at least in Web 1.0-related tasks [29]. Third, the envisioned tool could test less the skill of accessing (where the variance is low) and test more the advanced skills. Fourth, tasks will need to be more structured to allow for automatic scoring that does not rely on complex evaluation.

### Conclusions

A better understanding and assessment of eHealth literacy is essential in order to improve ICT use for health purposes by ordinary citizens. Improved understanding and assessment are prerequisites for enhancing eHealth literacy, thereby empowering patients in self-management of their health. This is even more important to those needing this most, such as long-term patients and the elderly. The present study demonstrated that performed eHealth literacy could be validly and reliably measured, that it is related to both human observations of skill, motivation, confidence, provision of help, and background characteristics, on the one hand, and to self-perceived eHealth literacy, on the other hand. The next stage of developing computerized adaptive short testing tools for eHealth literacy is advocated.

## Appendix

Multimedia Appendix 1 Specific tasks.

Multimedia Appendix 2 Coding scheme of performance by skill type for digital and electronic health literacy skills.

## Abbreviations

eHEALS: eHealth Literacy Scale
eHealth: electronic health
ICT: information and communication technology
