# Supplementary material for: Perceived and Performed eHealth Literacy: Survey and Simulated Performance Test
Source: JMIR Hum Factors. 2017 Jan 17;4(1):e2. doi: 10.2196/humanfactors.6523 (PMC5285606; doi:10.2196/humanfactors.6523)
Supplement: Multimedia Appendix 1 [file humanfactors_v4i1e2_app1.pdf]

Welcome to the Internet Use and Health Simulation

In the course of this study, you will be asked to complete 15 tasks; each task is allotted a different amount of time.

Please note: before the beginning of each task you need to press the START button, and once you have completed the task, press the END TASK button.

At this stage, if something is unclear, please ask me. When I indicate, you can press the START button and begin your first task.

Your cooperation is greatly appreciated!

The Research Team

The Tasks

First Set of Tasks

Time allotted– maximum of 12 minutes

1. Please choose a browser (you may choose from among various browsers, for example MS Explorer, Firefox, or others) from the computer desktop, and open it in a new window.
Choose a search engine (for example, Google).
Coded for: Medium-related, operational skill.

2. Open an additional tab in that window.
Coded for: Medium-related, operational skill.

3. Please type in the entry "Maccabi HMO" (Health Maintenance Organization) in the search engine box.

- Press the SEARCH button
- From the results provided, select the most appropriate (the Maccabi HMO website).
- Finally, add the homepage of the Maccabi HMO website to the *favorites* list on the computer.

Coded for: Medium-related, operational skill; access, eHealth literacy skill.

4. Return to the search engine's home page.

- Type 'Maccabi HMO' in the search box, press the SEARCH button and open the most appropriate result.

- Press the LINKS button in the 'Maccabi HMO' website
- Press the link to the Social Security Institute in the window that opens.
- Press the DOWNLOAD FORMS link in the new window of the Social Security Institute's website,
- Scroll down, using the mouse, until you find the menu for '*nursing care'*.
- Press the icon to open the PDF file titled *Request for Nursing Care Benefits (2006)*
- Save the downloaded form on the desktop.

Coded for: Medium-related, operational and information skills; access, eHealth literacy skill

5. Return to the' Maccabi HMO' website and increase the font size on the website, in order to make it easier to read.
Coded for: Medium-related, operational skill.

Tasks Related to Formal Skills
Second Set of Tasks
Time allotted – maximum of 15 minutes

6. Open the 'Maccabi HMO' website. You have three possible ways to do this.

a) Using a search engine.
b) Typing the name of the website in the address/search box in the browser window.
c) Using the link you saved in the *favorites* list.

Once you have reached the website, please find out the following: what are the rights related to medication purchasing to patients who have nursing care insurance provided by 'Maccabi Gold Plate' insurance program?
Tell me what you have discovered.
Coded for: Medium-related, formal skill; Content-related information skill; access, understand, and appraise eHealth literacy skills

7. Assume that you have taken a blood test and you wish to understand the meaning of the values written under the PLT test (also known as thrombocytes or platelets). Your task is to determine which values are considered normative for this test.
To this end:

- Go to a search engine (for example, Google)
- Type a relevant question into the search box.
- Choose one of the relevant links from those on the first page of results retrieved (for example Wikipedia, Doctors, etc.).
- Read the information regarding normative results for blood platelet tests.
  Explain to me what you found.

Coded for: Content-related information skill; access, understand, and appraise eHealth literacy skills

8. Find an additional website that provides information regarding the normative values for blood platelet tests, in order to verify the reliability of the information previously retrieved.
Explain how you would decide which information source is more reliable. What criteria do you use to determine reliability?
Coded for: Content-related information skill; access, understand, and appraise eHealth literacy skills

Tasks Related to Information Skills
Third Set of Tasks
Time allotted – maximum of 10 minutes

9. Choose one of the following topics:

- Shingles
- Vertigo
- Hearing Loss

Use the Internet to search for information on the topic of choice and describe what you have found.
Coded for: Content-related information skill; access, understand, and appraise eHealth literacy skills

Time allotted maximum of 15 minutes

10. Enter the YouTube website, then type the topic of choice from the previous task in the search box.

- Find relevant clips of up to 3 minutes' length.
- Open one of the clips and watch it.
- After you have watched it, please explain why you chose this particular clip.

Coded for: Content-related information skill; access, understand, and appraise eHealth literacy skills

Time allotted – maximum of 12 minutes

11. Imagine you have begun to experience knee pain. Please use your HMO website to find an orthopedic knee expert in your residential area and note the relevant times for scheduling an appointment (days and times).
Coded for: Content-related information skill; access, understand and apply eHealth literacy skills

12. Imagine you have experienced hearing loss in your right ear. Please search for available time slots for taking a hearing test through your local HMO center.
Coded for: Content-related information skill; access, understand and apply eHealth literacy skills

Time allotted – maximum of 12 minutes

13. Imagine you had an appointment with an orthopedic knee expert, who told you that your knee pain is a result of pressure on the knee and that your knee cartilage is damaged. Please find two possible symptoms of knee cartilage damage and explain them to me.
Coded for: Content-related information skill; access, understand and appraise eHealth literacy skills

Tasks Related to Strategic Skills

Fourth Task Set

Time allotted – maximum of 20 minutes

14. One day, after experiencing severe back pain, you decided to stay at home rather than go to work or to your community center.

- Using the Internet, find two types of treatments that can help relieve back pain.
- Which treatment would you choose as most appropriate?

Coded for: Content-related strategic skill; access, understand, appraise and apply eHealth literacy skills

Time allotted – maximum of 12 minutes

15. Find a website that includes a forum on health topics.
Find the appropriate place on the forum to ask about knee pain, give the symptoms you found in the previous task, and ask for advice about alternative treatments.
Coded for: Content-related operational and information skill; content-related strategic skill; access, understand, appraise apply and generate eHealth literacy skills

Thank you for your cooperation!
