# Supplementary material for: Perceived and Performed eHealth Literacy: Survey and Simulated Performance Test
Source: JMIR Hum Factors. 2017 Jan 17;4(1):e2. doi: 10.2196/humanfactors.6523 (PMC5285606; doi:10.2196/humanfactors.6523)
Supplement: Multimedia Appendix 2 [file humanfactors_v4i1e2_app2.pdf]

Task number and description	Digital skills (adapted from van Deursen 2012, [11])				eHealth literacy skills (adapted from Sorensen et al. 2013, [26])				
	Medium-related		Content related						
	Operational skills	Formal skills	Information skills	Strategic skills	Access	Understand	Appraise	Apply	Generate (Web 2.0)
1. OpenBrowserQ1, EnterSearchEngineQ1	✓								
2.AddBrowserTabQ2	✓								
3. InsertQuestionQ3, PressButtonQ3, AddFavoritesQ3 HMO	✓				✓				
4. InsertQuestionQ4, ClickLinksQ4, RollBrowserQ4, ClickPdfQ4, SaveFileQ4. HMO	✓	✓			✓				
5. EnlargeFontSizeQ5	✓								
6. OpenSiteThrougSearchEngineQ6, OpenSiteThrougInsertNameQ6, OpenSiteThrougFavoritesQ6. HMO		✓	✓		✓	✓	✓		

7. EnterSearchEngineQ7, InsertQuestionQ7. Google			✓		✓	✓	✓		
8. EnterSearchEngineQ8, InsertQuestionQ8. Google			✓		✓	✓	✓		
9. EnterSearchEngineQ9, InsertQuestionQ9. Google			✓		✓	✓	✓		
10. TypeTopicSearchBarQ10, OpenVideoQ10, WatchVideoQ10,			✓		✓	✓	✓		
11. EnterCorrectSiteQ11, FindTextBoxDataQ11, PressSearchQ11. HMO			✓		✓	✓		✓	
12. EnterCorrectSiteQ12, InsertQuestionQ12. HMO Google			✓		✓	✓		✓	
13. EnterSearchEngineQ13, InsertQuestionQ13. Google			✓		✓	✓	✓		
14. EnterSearchEngineQ14, InsertQuestionQ14. Google				✓	✓	✓	✓	✓	
15. EnterSearchEngineQ15, InsertQuestionQ15, OpenCorrectResultQ15, EnterForumQ15. Google	✓	✓		✓	✓	✓	✓	✓	✓
